# The 13C-Glucose Breath Test for Insulin Resistance Assessment in Adolescents: Comparison with Fasting and Post-Glucose Stimulus Surrogate Markers of Insulin Resistance

**DOI:** 10.4274/jcrpe.3260

**Published:** 2016-12-01

**Authors:** Jorge Maldonado-Hernández, Azucena Martínez-Basila, Alejandra Salas-Fernández, José R. Navarro-Betancourt, Mónica I. Piña-Aguero, Mariela Bernabe-García

**Affiliations:** 1 National Medical Center “Siglo XXI”, Mexican Social Security Institute, Medical Nutrition Research Unit, Mexico City, Mexico

**Keywords:** 13C-glucose breath-test, insulin resistance, oral glucose tolerance test, adolescents

## Abstract

**Objective:**

To evaluate the use of the ^13^C-glucose breath test (^13^C-GBT) for insulin resistance (IR) detection in adolescents through comparison with fasting and post-glucose stimulus surrogates.

**Methods:**

One hundred thirty-three adolescents aged between 10 and 16 years received an oral glucose load of 1.75 g per kg of body weight dissolved in 150 mL of water followed by an oral dose of 1.5 mg/kg of U-^13^C-Glucose, without a specific maximum dose. Blood samples were drawn at baseline and 120 minutes, while breath samples were obtained at baseline and at 30, 60, 90, 120, 150, and 180 minutes. The ^13^C-GBT was compared to homeostasis model assessment (HOMA) IR (≥p95 adjusted by gender and age), fasting plasma insulin (≥p90 adjusted by gender and Tanner stage), results of 2-h oral glucose tolerance test (OGTT), insulin levels (≥65 μU/mL) in order to determine the optimal cut-off point for IR diagnosis.

**Results:**

^13^C-GBT data, expressed as adjusted cumulative percentage of oxidized dose (A% OD), correlated inversely with fasting and post-load IR surrogates. Sexual development alters A% OD results, therefore individuals were stratified into pubescent and post-pubescent. The optimal cut-off point for the ^13^C-GBT in pubescent individuals was 16.3% (sensitivity=82.8% & specificity=60.6%) and 13.0% in post-pubescents (sensitivity=87.5% & specificity=63.6%), when compared to fasting plasma insulin. Similar results were observed against HOMA and 2-h OGTT insulin.

**Conclusion:**

The ^13^C-GBT is a practical and non-invasive method to screen for IR in adolescents with reasonable sensitivity and specificity.

**WHAT IS ALREADY KNOWN ON THIS TOPIC?**

The ^13^C-glucose breath test (13C-GBT) is an accurate and reliable method to detect glucose metabolism disorders in adults, however, the use of this technique to assess insulin resistance (IR) in pediatric individuals is still under examination.

**WHAT THIS STUDY ADDS?**

The ^13^C-GBT was evaluated in a large population that included individuals with different body mass indexes and stages of pubertal development, furthermore, the A% oxidized dose was compared against IR surrogates in both fasting and post-load scenarios. This protocol suggests cut-off points to identify IR with reasonable sensitivity and specificity.

## INTRODUCTION

Type 2 diabetes mellitus (T2DM) is a prevalent chronic disease and represents a grievous public health problem (1). This condition is initially asymptomatic, but it is a definite risk factor for cardiovascular disease, nephropathy, and neuropathy. Morbidity- and mortality-related T2DM decreases with adequate metabolic control, therefore, an early diagnosis is imperative (2). Insulin resistance (IR) is defined as a state in which a normal or elevated insulin level produces an attenuated biological response and constitutes a physiopathological basis for development of T2DM (3). IR is associated to a sedentary lifestyle and an unbalanced diet - risk factors commonly observed in adolescence. Thus, adolescence is a critical period of life for diagnosis and initiation of lifestyle intervention in at-risk individuals (4,5,6).

The gold standard to diagnose IR is the hyperinsulinemic euglycemic clamp (HEC) because it provides a direct, dynamic, and accurate assessment (7); nonetheless, this is an expensive and highly invasive method, unfeasible for standard clinical pediatric practice (8). Surrogate IR markers are frequently used to detect IR in ordinary settings, such as the homeostasis model assessment (HOMA-IR) which is based on fasting plasma glucose and insulin concentrations (9) and the Matsuda and DeFronzo (10) insulin sensitivity index (ISI-Composite) derived from plasma glucose and insulin levels throughout an oral glucose tolerant test (OGTT). These measurements have a moderate to good correlation with the HEC technique but remain invasive and poorly reproducible (11). The development of practical and non-invasive screening tests to detect IR is imperative (12).

The ^13^C-glucose breath test (^13^C-GBT) has been shown to be an accurate and reliable method to identify glucose metabolism disorders in adults (13,14). The ^13^C-GBT consists of ingestion of a 13C-glucose dose used as a tracer to label exhaled CO_2_ together with an oral load of non-labeled glucose to challenge insulin-dependent tissues. Patients with impaired glucose metabolism will have a reduced amount of exhaled ^13^CO_2_; this represents an indirect measurement of glucose oxidation via Krebs cycle (13,15). Lewanczuk et al (13) have shown that the ^13^C-GBT is effective to assess insulin sensitivity in obese individuals with T2DM. Recently, our group demonstrated that the ^13^C-GBT is a reproducible method to identify glucose metabolism defects also in adults without T2DM (14). We also established that the ^13^C-GBT is a valid method for screening for metabolic syndrome in adolescents (16).

The ^13^C-GBT has been extensively studied in adults. However, the use of this method to evaluate IR in pediatric individuals has been insufficiently explored. The purpose of this study was to assess the use of the ^13^C-GBT for IR detection in adolescents through comparison with fasting and post-glucose stimulus IR surrogates.

## METHODS

This cross-sectional study was conducted in the Medical Nutrition Research Unit of the Mexican Social Security Institute in Mexico City, Mexico. The protocol was approved by the Ethics Committee of the said institution (R-2010-3603-35). One hundred thirty-three apparently healthy adolescents aged between 10 and 16 years assented to participate in the study. Informed consent was provided by the parents or by the accompanying adult. Exclusion criteria were: current chronic disease, diagnosed T2DM or presence of a capillary blood glucose level of ≥126 mg/dL, the use of medications that affect glucose metabolism, and fever in the last 48 hours.

### Procedures

Voluntary participants arrived to the Medical Nutrition Research Unit with their parents or legal guardian at 8:00 am after an 8-hour fast. Anthropometric measurements (weight, height, and body mass index) were obtained. Subjects received an oral glucose load of 1.75 g per kg of body weight up to a maximum dose of 75 g (ACS reagent; Sigma-Aldrich, St. Louis, MO) dissolved in 150 mL of water, followed by a dose of 1.5 mg/kg of universally labeled ^13^C-glucose (Cambridge Isotope Laboratories, Inc., Andover, MA, USA) mixed in 50 mL of water, without a specific maximum dose. Blood samples were drawn at baseline and 120 minutes through an antecubital venipuncture. Breath samples were obtained at baseline and at 30, 60, 90, 120, 150, and 180 minutes in 10 mL Exetainer® test tubes (Labco Limited, UK), because in a previous protocol, ^13^C-GBT results had the highest reproducibility when measured at 180 minutes (14).

### Biochemical Determinations

Plasma glucose levels were quantified using an enzymatic method (YSI 2300 Stat Plus™ glucose analyzer; YSI Inc., Yellow Springs, OH), and plasma insulin levels were measured by radioinmmunoassay employing a commercial kit (Millipore, Billerica, MA). The coefficients of variation (CV%) for glucose and insulin were 3.9% and 7.5%, respectively.

As an IR surrogate, the HOMA-IR was calculated with the following formula (9):

HOMA-IR=[fasting glucose (mg/dL) *fasting insulin (µU/mL)]/405.

### Breath CO_2_ Measurements

Carbon 13 in breath samples was determined with an isotope ratio mass spectrometer BreathMat Plus (Finnigan, Bremen, Germany; CV <1%). Breath test data were expressed as cumulative percentage of oxidized dose at 180 minutes (A% OD) as described previously (17).

### Statistical Analysis

Data analysis was performed with the SPSS software (version 19; SPSS Inc., Chicago, IL, USA). Kolmogorov-Smirnov test was used to assess data distribution. Data are presented as mean ± standard deviation or median (minimum-maximum) for normal or non-normal distribution, respectively. ANOVA test with Tukey post-hoc analysis or Kruskall-Wallis tests with Mann-Whitney U-test were used for the comparison between groups according to data distribution. Linear multiple regression models were used to summarize associations of insulin resistance surrogates (HOMA-IR, fasting plasma insulin and 2-h OGTT insulin) with sexual development (Tanner stage) and gender. Correlation coefficients were determined with Pearson’s or Spearman’s analyses according to data distribution. To determine the optimal cut-off point for IR diagnosis through the ^13^C-GBT, several receiver-operating characteristic (ROC) curves were constructed with a 95% confidence interval.

## RESULTS

A total of 133 adolescents (62 females and 71 males) living in Mexico City were enrolled during 2011. Mean age was 13 years, weight and abdominal circumference values ranged from 34 to 113 kg and from 63 to 129 cm, respectively. Body mass index (BMI) presented a median of 23 (15.6 to 37.8 kg/m^2^), and this parameter was used to classify individuals into three groups according to the child growth standards established by the World Health Organization (18), namely, lean (BMI between p3 and p85, 42.1%), overweight (BMI >p85, 14.3%) and obese (BMI >p97, 43.6%). Data describing the study sample and the statistically significant differences between the subgroups are summarized in Table 1.

The following parameters had statistically significant differences among the three subgroups: weight, BMI, abdominal circumference, fasting plasma insulin, and HOMA-IR (19). When contrasting lean versus obese and overweight versus obese individuals, 2-h OGTT insulin and A% OD at 180 minutes differed significantly. The comparison of lean versus overweight and lean versus obese subjects revealed that the 2-h OGTT glucose was substantially different. Finally, fasting plasma glucose achieved a statistically relevant difference only between lean and obese individuals.

Three multiple regression models with three different IR surrogates were used to determine the influence of Tanner stage and gender on ^13^C-GBT; IR was defined as HOMA-IR ≥p95 reference score adjusted by gender and age (19), fasting plasma insulin ≥p90 reference score adjusted by gender and Tanner stage (20), and 2-h OGTT insulin ≥65 μU/mL (21). Gender does not substantially alter ^13^C-GBT when co-analyzed with HOMA-IR (β=0.8; p=0.361), fasting plasma insulin (β=1.0; p=0.239), and 2-h OGTT insulin (β=1.4; p=0.131). In contrast, it was established that Tanner stage modifies ^13^C-GBT when co-evaluated with HOMA (β=-2.1; p=0.017), fasting plasma insulin (β=-1.9; p=0.034), and 2-h OGTT insulin (β=-2.1; p=0.017), therefore, in subsequent analyses, the sample was stratified into pubescent (Tanner stages 2 and 3) and post-pubescent (Tanner stages 4 and 5) individuals. Of the total sample, 46.6% were classified as pubescent and 53.4% as post-pubescent.

A Spearman’s rank correlation coefficient revealed that BMI, HOMA-IR, fasting plasma insulin, 2-h OGTT insulin, and 2-h OGTT glucose were inversely associated to A% OD at 180 minutes (Table 2). In contrast, fasting plasma glucose did not achieve statistical significance.

**Figure 1:**
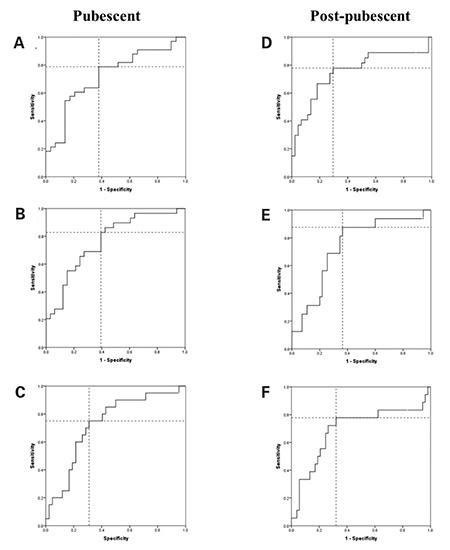
Receiver operating characteristic curves evaluating the sensitivitiy and specificity of the 13C-glucose breath test using different criteria to define insulin resistance. A-C: Performance in pubescent individuals. D-C: Performance in post-pubescent individuals. A, D= HOMA-IR ≥p95 reference score; B, E= fasting plasma insulin ≥p90 reference score; C, F: plasma insulin at the 2-hour time point of an oral glucose tolerance test ≥65 μU/mL

Several ROC curves were plotted to determine the optimal cut-off points for the A% OD at 180 minutes according to different IR surrogates as described previously (Figure 1). Diagnostic attributes of the ^13^C-GBT for each cut-off point are presented in Table 3. In pubescent and post-pubescent individuals, the ^13^C-GBT rendered the highest accuracy when compared to fasting plasma insulin. With said parameter, in pubescent individuals, an A% OD at 180 minutes ≤16.3% diagnoses IR with a sensitivity of 82.8%, a specificity of 60.6%, a positive predictive value (PPV) of 64.9%, and a negative predictive value (NPV) of 80.0%. In post-pubescent subjects, an A% OD at 180 minutes ≤13.0% indicates IR with a sensitivity of 87.5%, a specificity of 63.6%, a PPV of 41.1%, and a NPV of 94.6%.

## DISCUSSION

The purpose of this study was to explore the use of the ^13^C-GBT to identify IR in adolescents with different BMIs. Even though the use of ^13^C-GBT has been extensively described in adults, to our knowledge, definite cut-off points for pediatric populations have not yet been established. In this study, we compare the ^13^C-GBT with fasting plasma insulin, HOMA-IR, and 2-h OGTT insulin to propose several cut-off points for IR diagnosis. Similar values of sensitivity, specificity, PPV, and NPV were observed among the different IR surrogates contrasted with the ^13^C-GBT. Our results are similar to those described by Ibarra-Pastrana et al (22) in Mexican adults, where the ^13^C-GBT rendered a sensitivity of 80% and a specificity of 67.4% when compared to HOMA-IR. Moreover, the cut-off points proposed in this article are comparable with the ones recommended for the detection of metabolic syndrome in adolescents (sensitivity=81.5% and specificity=66.7%) in a recent study published by our group (16).

The ^13^C-GBT is based on the premise that subjects with impaired insulin sensitivity have a reduced glucose uptake in response to insulin; consequently, the A% OD at 180 minutes is diminished in subjects with IR. Indeed, the ^13^C-GBT correlates inversely with fasting and post load IR surrogates. Similar associations were described by Jetha et al (15) who compared the ^13^C-GBT with fasting plasma insulin (r=-0.51, p<0.01), HOMA-IR (r=-0.51, p<0.01) and 2-h OGTT insulin (r=-0.040, p<0.05) in 39 pre-pubescent obese individuals.

Interestingly, even though the A% OD at 180 minutes is inversely associated to BMI, HOMA-IR, fasting plasma insulin, 2-h OGTT insulin and 2-h OGTT, fasting plasma glucose is not statistically correlated, probably because fasting plasma glucose is affected tardily in the pathogenesis of T2DM (23), this supports employing the ^13^C-GBT for early IR detection.

Undoubtedly, a weakness in this protocol is that the ^13^C-GBT was not contrasted against the HEC which is considered the gold standard to assess insulin sensitivity, therefore, the suggested cut-off points may undervalue or overestimate the true diagnostic performance of the ^13^C-GBT. However, the HEC is highly invasive and relatively unsuitable for pediatric subjects, thus, the ^13^C-GBT was compared against commonly used IR surrogates. Future studies to validate the sensitivity and specificity of the ^13^C-GBT for IR in adolescents are warranted.

The evaluation of the ^13^C-GBT in a substantial sample with a wide BMI spectrum is a definite strength of this study. Even though obesity is strongly associated to IR, it is now accepted that physically lean subjects may have defective insulin sensitivity and are candidates for IR screening (24,25). Another valuable asset is that the proposed cut-off points for the ^13^C-GBT were stratified according to sexual development, since it has been established that sexual hormones influence glucose homeostasis (26,27).

Considering the increasing evidence regarding the existence of noxious metabolic disorders at young ages, the epidemiological relevance of T2DM, the long-term complications associated to impaired glucose homeostasis, and the knowledge that lifestyle interventions in individuals with IR are effective to prevent or delay the onset of T2DM (28), the development of methodologically feasible procedures to screen for IR in large populations is fundamental for the implementation and assessment of public health strategies to face the T2DM epidemic. The use of non-invasive methods to detect IR is particularly appealing for pediatric populations, where pain and emotional stress associated to venipuncture are continuing concerns (29). Moreover, T2DM is recognized as a progressive disease; in fact, a young age at onset of T2DM is associated with a higher incidence of macrovascular complications (30), therefore, early IR detection is distinctly relevant in pediatric subjects. The non-invasive nature of the ^13^C-GBT and its reasonable diagnostic performance render this method suitable to perform IR screening in large pediatric populations.

## Acknowledgments

The authors acknowledge BSc. Filiberto Jasso Saavedra for his technical assistance.

## Ethics

Ethics Committee Approval: This protocol was approved by the Ethics Committee of the Mexican Social Security Institute (registry number: R-2010-3603-35) in Mexico City, Mexico, Informed Consent: Informed consent was provided by the parents or by the accompanying adult.

Peer-review: External and Internal peer-reviewed.

## Figures and Tables

**Table 1:**
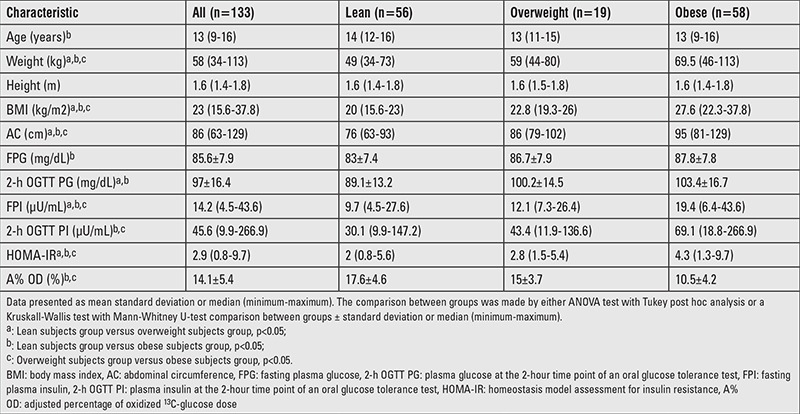
Inter-group comparison of the study group according to body mass index

**Table 2:**
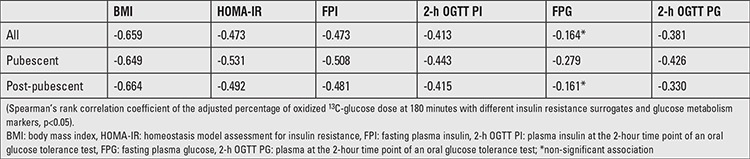
Correlation of the adjusted percentage of oxidized ^13^C-glucose dose at 180 minutes with different insulin resistance surrogates

**Table 3:**
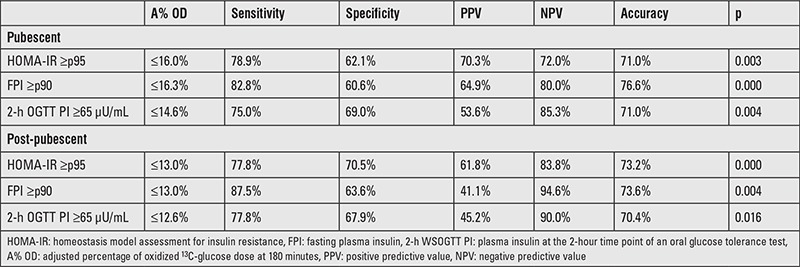
Diagnostic attributes of the ^13^C-glucose breath test (according to different cut-off points for the oxidized ^13^C-glucose dose at 180 minutes)
